# Pressure mat analysis of the longitudinal development of pig locomotion in growing pigs after weaning

**DOI:** 10.1186/1746-6148-10-37

**Published:** 2014-02-06

**Authors:** Ellen Meijer, Christian P Bertholle, Maarten Oosterlinck, Franz Josef van der Staay, Willem Back, Arie van Nes

**Affiliations:** 1 Department of Farm Animal Health, Faculty of Veterinary Medicine, Utrecht University, Yalelaan 7, NL-3584 CL Utrecht, The Netherlands; 2 Department of Surgery and Anaesthesiology, Faculty of Veterinary Medicine, Ghent University, B-9820 Merelbeke, Belgium; 3 Department of Equine Sciences, Faculty of Veterinary Medicine, Utrecht University, Yalelaan 112-114, NL-3584 CM Utrecht, The Netherlands

**Keywords:** Kinetics, Gait analysis, Pig, Symmetry

## Abstract

**Background:**

Gait evaluation is difficult in pigs, especially when objective and quantitative data are needed, thus little research has been conducted in this species. There is considerable experience, however, with objective gait analysis in other species, such as horses and dogs. In this study, a pressure mat was used to establish baseline kinetic data for gait and its longitudinal development in growing, weaned piglets.

Ten clinically healthy weaned piglets were trained to trot over a pressure mat. Measurements were performed weekly during 10 weeks, starting at 5 weeks of age. Four kinetic parameters were recorded for all four limbs: peak vertical force (PVF), load rate (LR), vertical impulse (VI) and peak vertical pressure (PVP). Three representative runs per measuring session per pig were collected. For each of the variables, left vs. right limb asymmetry-indices (ASI’s) were calculated based on the average for that parameter per week. A linear mixed model was used to determine the influence of time (week), velocity, and limb (left vs. right, and fore vs. hind). Intra-class correlations were calculated to assess within-session replicability.

**Results:**

Intra-class correlations showed good within-session replicability. Body-weight normalized PVF (nPVF), LR (nLR), VI (nVI) and PVP (nPVP) were higher in the forelimbs than in the hind limbs. A higher velocity was associated with a higher nPVF, nLR and nPVP. All parameters varied between weeks. ASI of LR and VI were higher in the forelimbs than in the hind limbs. Velocity and time did not influence ASI of any of the variables.

**Conclusions:**

Kinetic pressure mat measurements from healthy weaned piglets are highly replicable within-session. However, these variables present a significant variability between-session, which may be due to conformational changes of the young, growing piglets. Velocity clearly influences nPVF, nLR and nPVP, and all kinetic variables have higher values in forelimbs than in hind limbs. As time and velocity do not affect ASI’s, the latter are preferable tools when velocity cannot be controlled or when measurements are repeated over longer time intervals. The present study supports the use of a pressure mat as an objective way to analyze and quantify porcine gait.

## Background

Lameness is an important problem in modern swine husbandry. Prevalence of lameness in a cross-sectional study in the United Kingdom was estimated to be 14.4% in pregnant gilts, 16.9% in pregnant sows and 19.7% in finishing pigs [1]. Lameness has negative consequences from an animal welfare as well as from an economic point of view. The negative consequences of lameness on animal welfare are primarily due to pain and the resulting reduced mobility. A lame pig may not be able to reach feeding and drinking facilities and at the same time has a higher risk to be overrun by penmates, encountering additional trauma further reducing its welfare. The economic impact of lameness is caused by lower productivity and higher costs of treatment or even early culling of affected animals [2,3].

To minimize the aforementioned negative consequences of lameness and increase the chances of recovery it is critical to detect lame pigs as early as possible. Subtle changes in posture or weight bearing may occur in early stages of the disease process, and can easily be missed when gait is only assessed visually [4]. Furthermore, these changes can easily be overlooked in a pen with many pigs. Fast, sensitive, yet practical methods to detect lameness are necessary to help farmers to provide timely care for lame pigs, and to adequately measure the effect of interventions. Therefore, an objective method that does not only identify lame pigs but also quantifies the degree of lameness is needed to provide evidence-based information.

Several techniques have been developed for this purpose. The simplest and least expensive methods are lameness scoring systems based on visual inspection. The visual lameness scoring system for finishing pigs developed by Main et al. [5] incorporates gait characteristics (weight bearing on lame limb, stride length, caudal body sway), posture and behavior (both in response to humans and within the group of animals). Although visual scoring is fast and inexpensive, research in horses and dogs has shown that it may suffer from inherent subjectivity, is affected by observer bias and has limited intra- and inter-rater agreement, especially in untrained observers and in mild lameness [5-9]. Similarly, visual grading of mild lameness in pigs has been proven to be subjective [4]. These drawbacks underline the need for a more objective method to quantify lameness.

Kinematic techniques have been used in pigs to study the effect of different flooring types on locomotion [10,11] and to quantify lameness in sows [12], although this rather complicated, expensive and time-consuming methodology is unlikely to be extrapolated to a practical situation. Therefore, in pigs as well as in other species, force plates have become ‘the gold standard’ to objectively evaluate kinetic gait variables. They have been used in pigs to study the effect of different flooring types on gait kinetics [10] and to assess lameness in sows [13]. A major drawback in the use of force plates is that they cannot distinguish between several feet simultaneously in contact with the plate. Therefore, data collection can be time-consuming, or multiple consecutive force plates are needed to gather data of all limbs. The two parameters most often used in force plate analysis, peak vertical force (PVF) and vertical impulse (VI) are strongly influenced by velocity [14-16]. Therefore, velocity needs to be controlled within strict limits if footfalls from different runs are to be compared to each other. This might be a problem in pigs, since they are difficult to handle and to guide over the runway at a certain pace.

Pressure mats may provide a solution to this problem, as they contain a dense array of pressure sensors with a high measuring frequency, enabling them to distinguish simultaneous impacts of different limbs. This equipment allows measuring kinetic as well as spatiotemporal data of simultaneous and even consecutive footfalls. Systems of different manufacturers have been used successfully to evaluate locomotion in sound horses [17,18], cows [19], sheep [20], dogs [21], and cats [22], and to assess lameness in dogs [23,24] and cows [25]. Previous studies have shown that pressure-measuring systems may be useful to study the pressure distribution within each claw [26] and to measure PVF symmetry in an experimental lameness model in sows [27]. However, comprehensive baseline data describing the replicability, longitudinal development, and major confounding effects on pressure mat variables in weaned piglets are lacking.

Therefore, the aim of this experiment was to investigate the use of a pressure mat to evaluate longitudinal development of locomotion in growing, weaned pigs. Multiple pressure mat measurements were performed weekly during 10 weeks, starting at the age of 5 weeks. We evaluated the replicability of body-weight normalized peak vertical force (nPVF), load rate (nLR), vertical impulse (nVI) and peak vertical pressure (nPVP) as well as asymmetry indices (ASI’s) of these variables, to establish baseline data for pressure mat analysis in growing pigs.

## Results

**Figure 1:**
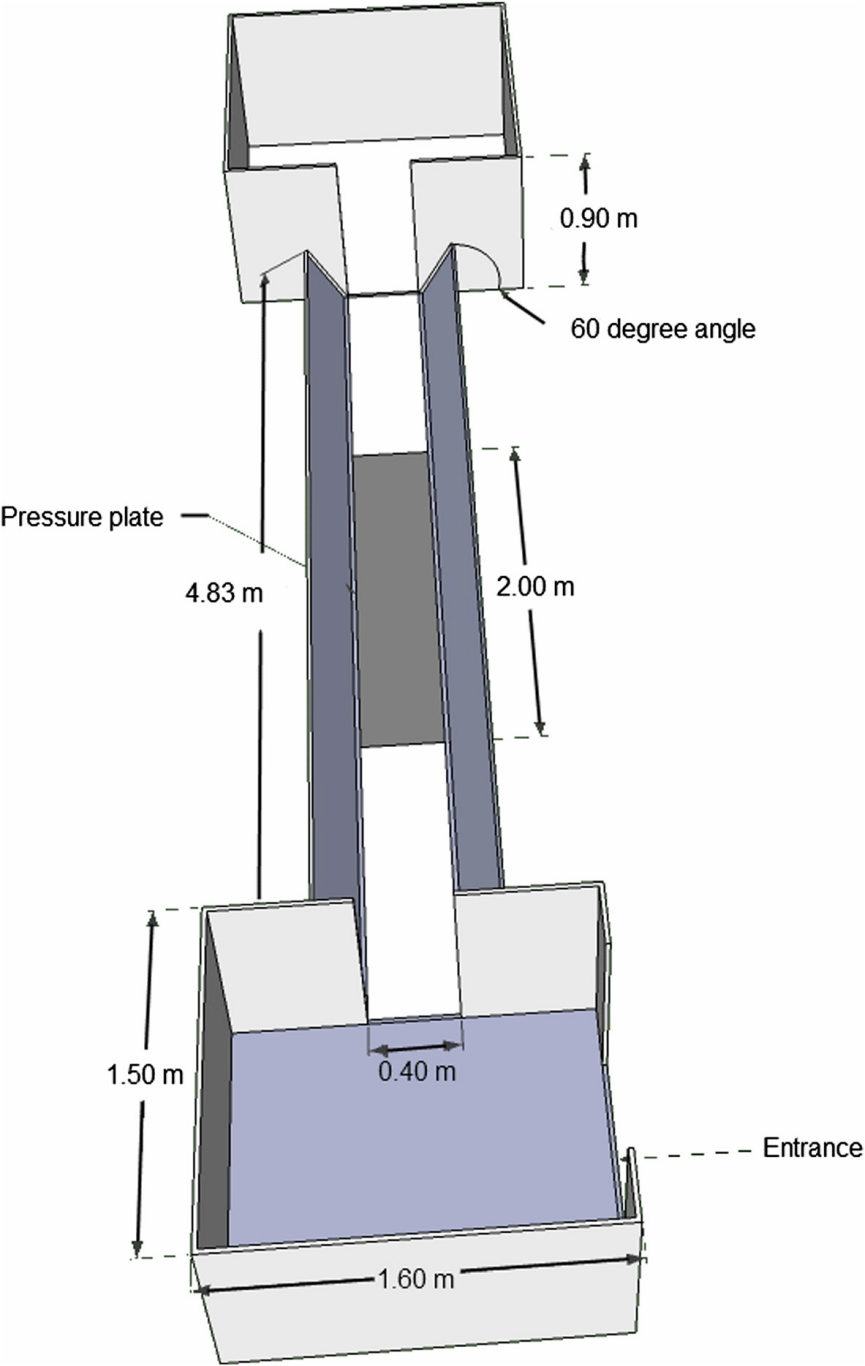
Schematic illustration of the experimental setup, including the runway containing a pressure mat.

**Figure 2:**
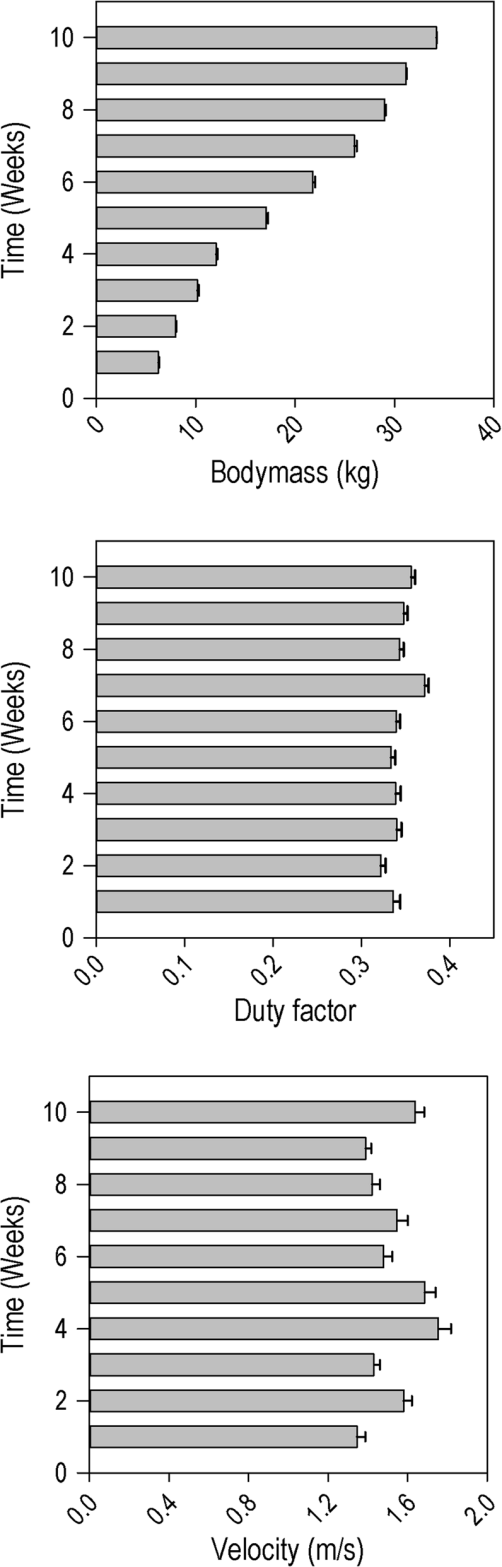
Velocity (mean ± SEM over all pigs, upper left panel), bodymass (mean ± SEM over all pigs, upper right panel) and duty factor (mean ± SEM over all pigs, lower left panel) for each week of the study period.

At the start of the experiment, pigs’ body mass was 6.25 ± 0.06 kg. At the end of the experiment, 10 weeks later, their body mass had increased to 34.2 ± 0.07 kg. The overall mean velocity of valid runs was 1.53 ± 0.01 m/s. The experimental setup is illustrated in Figure 1. Bodymass, velocity and duty factor per week are summarized in Figure 2.

### Pathology

The limb joints did not show any macroscopic abnormalities. Longitudinal cuts through the humerus, radius, ulna, femur, tibia, and fibula were macroscopically normal. No abnormalities were noted in skin, muscles, or connective tissue in any of the limbs. Therefore, all pigs were considered to be sound and healthy at scheduled necropsy.

### Replicability

Intra-class correlations (ICC) between runs on the same day were fair to excellent. ICC for nLR was the lowest (0.644), followed by the ICC for nPVF (0.802) and nPVP (0.858). ICC for nVI was the highest (0.881).

### Pressure mat variables

Average pressure mat variables per limb are summarized in Table 1.

**Table 1:** Pressure mat gait variables of trotting pigs (mean ± SEM) over all pigs and over the complete study period

**Variable**	**Left fore**	**Right fore**	**Left hind**	**Right hind**
Weight distribution (%)	0.29 ± 0.00	0.29 ± 0.00	0.21 ± 0.00	0.21 ± 0.00
nPVF (N/kg)	6.67 ± 0.09	6.61 ± 0.09	4.83 ± 0.09	4.93 ± 0.08
PVF (% BW)	67.99 ± 0.88	67.33 ± 0.92	49.24 ± 0.87	50.23 ± 0.78
nLR ((N/s)/kg)	0.12 ± 0.00	0.12 ± 0.00	0.11 ± 0.00	0.11 ± 0.00
nVI (Ns/kg)	0.67 ± 0.02	0.65 ± 0.01	0.46 ± 0.01	0.47 ± 0.01
VI (s*% BM)	6.85 ± 0.15	6.65 ± 0.14	4.72 ± 0.12	4.76 ± 0.12
nPVP((N/cm^2^)kg)	0.31 ± 0.01	0.30 ± 0.01	0.26 ± 0.01	0.26 ± 0.00

### Peak vertical force

**Figure 3:**
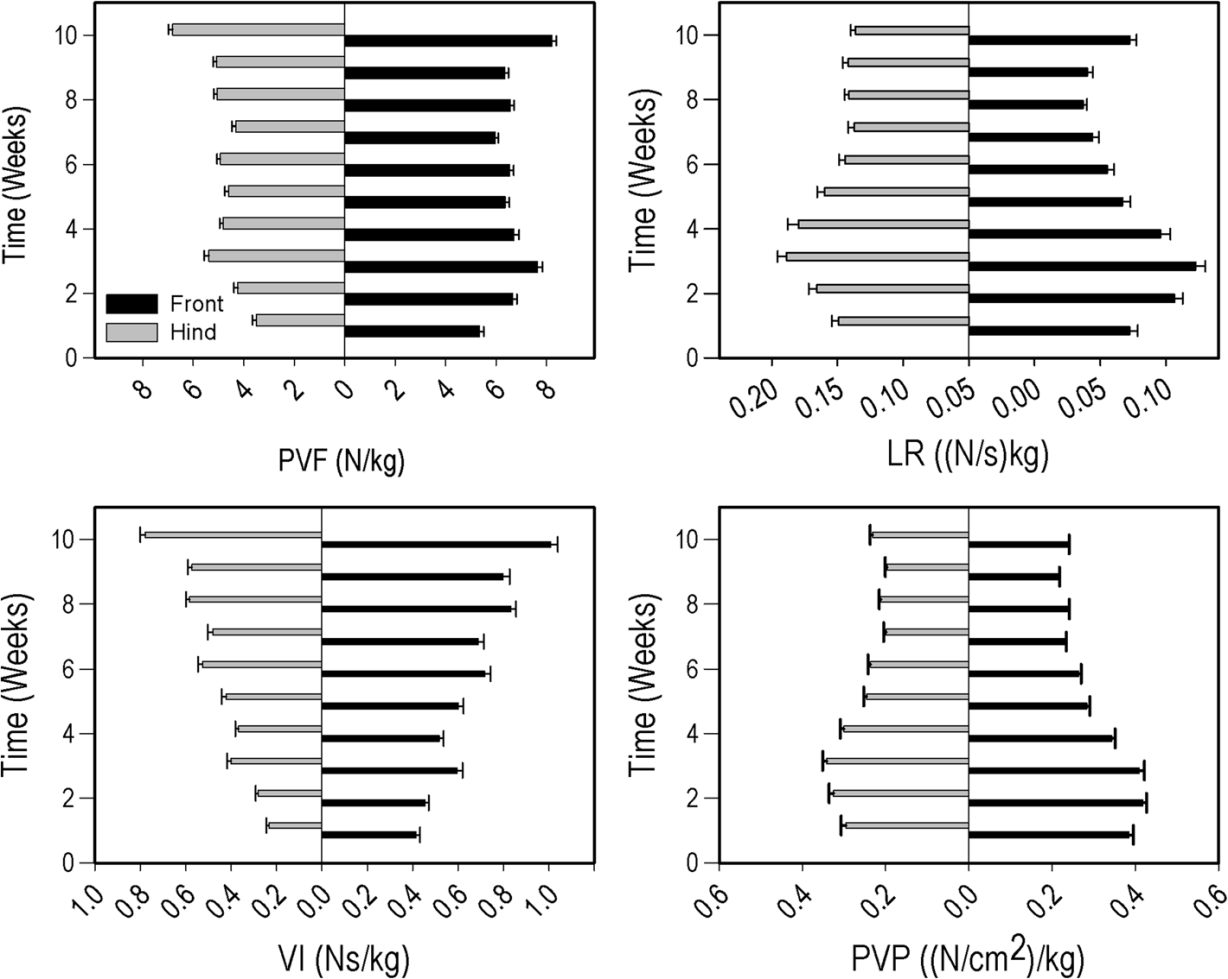
**Fore- and hind limb pressure mat variables (mean ± SEM over all pigs) for nPVF (upper left panel), nLR (upper right panel), nVI (lower left panel) and nPVP (lower right panel) for each week of the study period.** Legend in upper left panel applies to all panels. Significant differences between weeks are summarized in Tables 3, 4, 5, 6.

nPVF was affected by velocity F (1, 1175) = 31.73, P < 0.05), and was different between fore vs. hind limb (F (1, 1175) = 638.07, P < 0.05), and between the different time points (F (9, 1175) = 40.27, P < 0.05), but the direction of this influence differed between weeks (Figure 3, Table 2).

**Table 2:** Mean differences in nPVF by week controlling for velocity and fore-or hindlimb

**Week**	**1**	**2**	**3**	**4**	**5**	**6**	**7**	**8**	**9**	**10**
1	0.00	-0.95*	-2.05*	-1.23*	-0.96*	-1.27*	-0.66*	-1.36*	-1.27*	-3.01*
2		0.00	-1.10*	-0.28	-0.01	-0.32*	0.30	-0.41*	-0.32*	-2.05*
3			0.00	0.82*	1.09*	0.78*	1.40*	0.69*	0.78*	-0.95*
4				0.00	0.27	-0.04	0.58*	-0.13	-0.04	-1.77*
5					0.00	-0.31	0.31	-0.40*	-0.31	-2.04*
6						0.00	0.62*	-0.09	0.00	-1.73*
7							0.00	-0.71*	-0.61*	-2.35*
8								0.00	0.09	-1.64*
9									0.00	-1.73*
10										0.00

Numbers in table are column 1- row 1 difference. An *indicates a significant difference.

### Load rate

nLR was higher with increasing velocity (F (1, 1175) = 65.41, P < 0.05) and was higher in forelimbs than in hind limbs (F (1, 1175) = 25.19, P < 0.05). Time affected nLR (F (9, 1175) = 26.20, P < 0.05) but the direction of this influence varied between weeks (Figure 3, Table 3).

**Table 3:** Mean differences in nLR by week controlling for velocity and fore-or hindlimb

**Week**	**1**	**2**	**3**	**4**	**5**	**6**	**7**	**8**	**9**	**10**
1	0.00	-0.02*	-0.04*	-0.02*	0.00	0.01*	0.02*	0.02*	0.02*	-0.01
2		0.00	-0.02*	0.00	0.02*	0.04*	0.05*	0.04*	0.04*	0.01*
3			0.00	0.02*	0.05*	0.06*	0.07*	0.07*	0.06*	0.03*
4				0.00	.023*	.033*	.044*	.043*	.041*	0.01
5					0.00	0.01	0.02*	0.02*	0.02*	-0.01*
6						0.00	0.01	0.01	0.01	-0.02*
7							0.00	0.00	0.00	-0.03*
8								0.00	0.00	-0.03*
9									0.00	-0.03*
10										0.00

Numbers in table are column 1- row 1 difference. An *indicates a significant difference.

### Vertical impulse

Forelimb nVI was higher than hind limb nVI (F (1, 1175) = 570.17, p < 0.05) and generally increased over time (F (9, 1175) = 71.33, p <0.05) (Figure 3, Table 4). The effect of velocity was present in the initial analysis (p = 0.04), but was not significant after Bonferroni correction.

**Table 4:** Mean differences in nVI by week controlling for velocity and fore-or hindlimb

**Week**	**1**	**2**	**3**	**4**	**5**	**6**	**7**	**8**	**9**	**10**
1	0.00	-0.04*	-0.17*	-0.12*	-0.19*	-0.30*	-0.26*	-0.38*	-0.36*	-0.57*
2		0.00	-0.13*	-0.07*	-0.14*	-0.25*	-0.22*	-0.34*	-0.32*	-0.53*
3			0.00	0.06*	-0.01	-0.12*	-0.09*	-0.21*	-0.19*	-0.40*
4				0.00	-0.07*	-0.18*	-0.15*	-0.27*	-0.24*	-0.45*
5					0.00	-0.11*	-0.08*	-0.20*	-0.17*	-0.38*
6						0.00	0.03	-0.09*	-0.06*	-0.27*
7							0.00	-0.12*	-0.10*	-0.31*
8								0.00	0.02	-0.19*
9									0.00	-0.21*
10										0.00

Numbers in table are column 1- row 1 difference. An *indicates a significant difference.

### Peak vertical pressure

nPVP increased with higher velocity (F (1, 1175) = 22.67, p < 0.05). Forelimb nPVP was higher than hind limb nPVP (F (1, 1175) = 164.59, p < 0.05). Time influenced nPVP (F (9, 1175) = 92.71, p <0.05), and nPVP showed a tendency to decrease over time (Figure 3, Table 5).

**Table 5:** Mean differences in nPVP by week controlling for velocity and fore-or hindlimb

**Week**	**1**	**2**	**3**	**4**	**5**	**6**	**7**	**8**	**9**	**10**
1	0.00	-0.03*	-0.03*	0.02*	0.08*	0.09*	0.13*	0.12*	0.14*	0.11*
2		0.00	-0.01	0.05*	0.11*	0.12*	0.16*	0.15*	0.16*	0.14*
3			0.00	0.06*	0.11*	0.13*	0.16*	0.15*	0.17*	0.14*
4				0.00	0.06*	0.07*	0.10*	0.09*	0.11*	0.09*
5					0.00	0.01	0.05*	0.04*	0.06*	0.03*
6						0.00	0.04*	0.03*	0.04*	0.02*
7							0.00	-0.01	0.01	-0.02*
8								0.00	0.02*	-0.01
9									0.00	-0.03*
10										0.00

Numbers in table are column 1- row 1 difference. An *indicates a significant difference.

### Asymmetry indices

**Figure 4:**
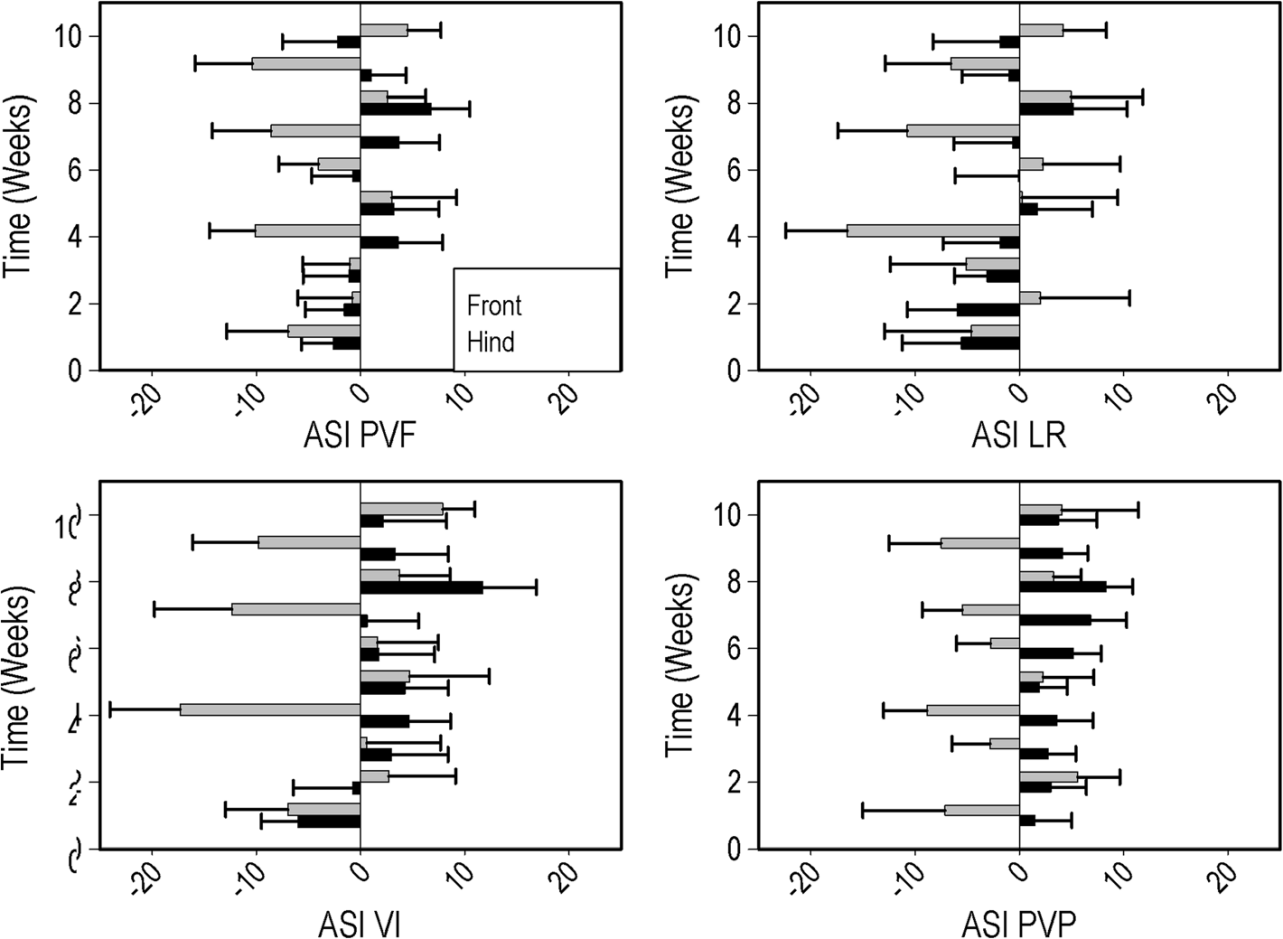
**Absolute values for forelimb and hindlimb ASI’s (mean ± SEM over all pigs) for PVF (upper left panel), LR (upper right panel), VI (lower left panel) and PVP (lower right panel) for each week of the study period.** Legend in upper left panel applies to all panels.

Average ASI’s for all parameters are summarized in Table 6. Forelimb ASI’s were higher than hind limb ASI’s for nLR (F (1,189) = 5.73, P < 0.05) and nVI (F (1,189) = 5.74, P < 0.05). This difference between fore- and hind limb ASI was also present in the initial analysis of nPVF (P = 0.03) and nPVP (P = 0.04), but was not significant after Bonferroni correction. Time did not have a significant effect on any ASI (Figure 4).

**Table 6:** Fore and hind limb absolute values for ASI’s (mean ± SEM) for nPVF, nLR, nVI and nPVP over all pigs in the complete study period

**Variable**	**Fore**	**Hind**
ASI PVF	0.97 ± 1.23	-3.19 ± 1.57
ASI LR	-1.23 ± 1.60	-2.98 ± 2.27
ASI VI	2.44 ± 1.54	-2.52 ± 2.05
ASI PVP	4.02 ± 0.95	-1.92 ± 1.44

## Discussion

This study is the first to explore the use of a pressure mat for longitudinal measurement of porcine locomotion and to establish baseline data for pressure mat analysis in weaned pigs. The data collection with the pressure mat was fast and efficient, and a maximum of 10 minutes per pig was needed to collect 3 valid runs. Training of the pigs proved to be simple and effective. The preparation of data for analysis, however, was very time-consuming, mainly because the software used in this study is designed for human gait analysis, and as such could not automatically distinguish the 4 limbs of the pigs. All footprints had to be assigned manually. Some other pressure mat manufacturers (for example Tekscan®) do provide software that can distinguish the 4 limbs of, for example dogs, automatically.

### Replicability

Intra-class correlations for pressure mat kinetic variables showed that data within one day were highly replicable, which is in agreement with a previous study in ponies by Oosterlinck et al. [18] reporting a high replicability of nPVF and nVI, and a slightly lower replicability of nPVP. Oosterlinck et al. [18] hypothesized that the higher variability in nPVP (which is force per unit of area) might have been due to limitations in the dynamic response of the activated sensors in the pressure mat.

### Absolute values of pressure mat kinetic variables

Mass-normalized forelimb nPVF was considerably lower in trotting pigs than in trotting ponies [18,28], horses [29] and dogs [30]. Mean hind limb nPVF was also lower than found in dogs [30]. nVI of the front limbs was lower than found in dogs [30] and ponies [18]. Some of these differences may be associated with differences in measuring equipment setup, such as measuring frequency and calibration. The relatively low measuring frequency in our study (126 Hz) may have caused some data points to be missed. However, this explanation is unlikely considering the mean stance time of a pig in our study (176 ms). We still have an average of 22 data points per curve, resulting in a smooth force graph. Also, the calibration procedures for pressure mats vary between studies, which may make comparison of variables more difficult. When comparing pressure mat data to force plate data, it has been shown in horses that a pressure mat cannot be used interchangeably to a force plate to measure absolute values of limb loading as it has limitations in accuracy [29], especially at impact and breakover [31]. The dynamic response of the pressure-measuring sensors may be slower than that of the piëzo-electric measurements of force plates, as was suggested by Besancon et al. [32] and Oosterlinck et al. [29].

The nPVP of the front limbs was higher than that found in ponies [18]. Since the nPVF was lower than in ponies, the magnitude of the nPVF cannot be the reason for the higher nPVP. Since nPVP is influenced by both nPVF and contact area, it may be that the contact properties of pig hooves promote higher nPVP. Further investigations of the nPVF and nPVP in different areas of the porcine hoof, using a pressure mat, may provide more information on this subject.

Little research is available on nLR, making this parameter hard to compare to other studies.

### Effect of velocity

In this study, pigs were trained to trot across the runway at a steady pace. In studies in other species, such as dogs, horses and sheep the animals were often led by a handler. The advantage of leading the animals across the runway is that it will take less time to collect valid runs, since the animal can be guided to walk in a straight line, at a steady pace and within certain speed limits.

Since pigs resist to being handled by a collar, another method had to be used. Training the pigs to trot over the runway without any form of guidance made sure the pig was trotting in a natural pattern and looking straight ahead, not turning their head or looking up or down.

Because the pigs were running without any guidance, the velocity of the pigs could not be strictly controlled. Still, the spread of the velocities was not very large. Velocity was measured by the pressure mat. In a previous study in dogs, pressure mat and photoelectric switch measurements of velocity yielded highly similar results [30]. Velocity significantly influenced nPVF, nLR and nPVP. The influence of velocity on nPVF is in agreement with previous reports in other species [14,17,29-33]. In the hind limbs of walking and trotting dogs, an increase of velocity is associated with a higher nPVF and a lower nVI [33,34]. Surprisingly, in the pigs we did not find a significant relationship between velocity and nVI. Vertical impulse is the amount of force applied over a certain amount of time (the duration of the step) and therefore depends on the stance time and the vertical force. Normally, with increasing speed the decrease in stance time is relatively more pronounced than the increase in nPVF, resulting in a decrease in nVI. In the present study, an increase of 1 m/s in speed caused a 0.31 N/kg increase in nPVF. It is unclear whether this effect may have been large enough to outweigh the effect of decreasing stance time with speed.

There is little information on the effect of velocity on nLR in quadrupeds. McLaughlin et al. [34] did, however, show that with increasing velocity, nPVF increases and stance time decreases in both horses and dogs. This could explain the effect of velocity on load rate, since a higher nPVF has to be achieved during a shorter time period.

In the present study, contact area was not affected by velocity. As nPVP is force per unit of area, it is possible that at higher velocities nPVP increases due to increasing nPVF while contact area remains constant.

### Effect of time

In this study, a significant difference between longitudinal measurements of mass-normalized nPVF, nLR, nVI and nPVP was found. It is known that besides speed, inter-trial variability is the most important confounding factor [35]. Lascelles et al. [30], however, did not find significant differences in pressure mat values for PVF and VI in measurements made 1 week apart in clinically normal mixed-breed dogs. Importantly, the latter studies were performed in adult dogs. In the present study, young, growing piglets were used, and even though the kinetic data were corrected for body mass, conformational changes occurred over the study period. Breed-dependent differences in kinetic data have been shown in dogs [36,37] and horses [38]. Thus, it seems possible that conformational changes in growing piglets may account for part of the longitudinal variation, especially because we followed the piglets for a long period of time compared to the studies by Mölsa et al. [36], Voss et al. [37] and Back et al. [38].

### Differences between fore- and hind limb

Our results on the difference in fore- and hind limb data for nPVF are in agreement with data in other species with a reported distribution of bodyweight of approximately 30% on each front limb and 20% on each hind limb [32,39,40].

### Asymmetry indices

Symmetry is generally assumed to be a characteristic feature of normal locomotion [18,41-44], whereas a substantial lack of symmetry usually correlates with the presence of pathology/lameness [23,24,45]. The trot is a symmetric gait, facilitating comparison of left vs. right limbs. Our results indicate a very low degree of asymmetry in sound pigs. However, some degree of asymmetry can even be observed in sound individuals [18,46]. Similar as reported in dogs [24], the 2 m-pressure mat in the present study allowed the recording of contra-lateral and consecutive foot strikes, and therefore, ASI between contra-lateral limbs were not affected by inter-trial variability. Notwithstanding the fact that the range of ASI observed in the present study was larger than the degree of (a)symmetry reported in dogs [24] and ponies [18], there were no significant differences in ASI over a prolonged time. Therefore, ASI are a highly promising tool for the longitudinal analysis of locomotion, prospecting evidence-based evaluation of lameness, effects of treatments etc. Cut-off values of ASI obtained from pressure mat analysis to distinguish lame and sound pigs have not yet been determined, but the present study provides normative data for ASI’s in young, sound pigs.

In agreement with previous work by Oosterlinck et al. [18], our results did not present a significant influence of velocity on ASI’s. This is particularly interesting for the measurement of gait in pigs, since in this species it is difficult to maintain a fixed speed over several trials without disturbing the natural gait of the animal. From a practical point of view, for this type of pressure mat further development of software for use in quadrupeds is needed. In order to facilitate the analysis and subsequent interpretation of kinetic symmetry in a clinical situation using this particular kind of pressure mat, automated selection and calculation of symmetry ratios would be interesting. The software currently available allows the automated selection of human feet, whereas in our study, manual selection of each footprint was needed. In large datasets, this may be time consuming and therefore the automated allocation of left/right fore and hind hoof prints in combination with automated calculation of (a-)symmetry indices between contra-lateral, ipsilateral and diagonal limb pairs would facilitate a swift interpretation of kinetic data.

## Conclusions

The present study provides normative kinetic data for young, sound pigs. Based on the significant effects of velocity, fore vs. hind limb, and measuring session on absolute values of kinetic variables, it is advised to set limits for speed. Moreover, measurements that are set apart in time (e.g. intervention studies) should be interpreted cautiously, especially in young growing pigs in which conformation may change. Fore- and hind limbs present different absolute values of limb loading and this must be accounted for when interpreting results.

In the present study, the pressure mat allowed recording contra-lateral and consecutive foot strikes. ASI’s of contra-lateral limbs were shown to have excellent replicability over time, and were not affected by speed. Therefore, we recommend the use of ASI of kinetic variables in further studies focusing on the discrimination between lame and sound pigs, the early detection of lameness, and the evidence-base evaluation of treatments.

## Methods

The study was reviewed and approved by the ethics committee of Utrecht University, The Netherlands, and was conducted in accordance with the recommendations of the EU directive 86/609/EEC. All effort was taken to minimize the number of animals used and their suffering.

### Animals

Ten 4-week-old healthy and sound Topigs 20 pigs (6 boars, 4 sows) were randomly selected from a commercial breeding farm. The pigs were transported to the animal facility of the Veterinary Faculty, Department of Farm Animal Health, Utrecht University.

### Housing

The pigs were housed in the research facility of Utrecht University. They were randomly divided over two pens with closed concrete floors, each pen measuring 153 cm × 256 cm. The ambient temperature in the stalls was 24°C. Two extra heat lamps per pen were provided during the first 6 weeks of the experiment. The piglets were exposed to both daylight and artificial lighting from 7 a.m. to 6 p.m. (11 hours a day). They had ad libitum access to water and food (Groeiporco, De Heus Animal Nutrition, Ede, The Netherlands). The pens were provided with toys (metal chain, plastic ball) during the entire experiment.

### Data recording

Every week, body mass was recorded using a weighing scale (MS Schippers, Bladel, The Netherlands), they were visually evaluated for lameness by a veterinarian using a scoring system modified from De Koning et al. [47], and pressure mat analysis was performed. The pressure mat was a Footscan® 3D Gait Scientific 2 m system (RSscan International, Olen, Belgium) with an active sensor surface of 1.95 m × 0.32 m containing 16384 sensors (2.6 sensors per cm^2^), with a sensitivity of 0.27-127 n/cm^2^ and a measuring frequency of 126 Hz, connected to a laptop with dedicated software (Footscan Scientific Gait 7 gait 2^nd^ generation, RSscan International, Olen, Belgium). Calibration of the pressure mat was performed according to the manufacturer’s instructions using a person weighing 70 kg. The mat was mounted flush with a 483 cm × 40 cm walkway. The entire walkway was covered with a 0.5 mm rubber mat (shore value 65° ± 5). To prevent the pigs from leaning against the wall and inadvertently influencing the measurements, the sides of the runway were inclining outward in a 60° angle. A 160 cm × 150 cm holding pen that could be closed was located at both ends of the runway (Figure 1). Velocity was measured by the pressure plate.

### Procedure

After the piglets arrived at the facility, they were allowed to acclimatize to the new environment for one week. On day 1, 3, and 5 of this first week, the pigs were habituated to the test apparatus and trained to trot over the pressure mat, using treats as reward when the animal had trotted over the runway without stopping. The training ended after the piglet had performed 3 correct runs. A training session was never longer than 10 minutes, so even after multiple unsuccessful attempts, after 10 minutes the piglets were returned to their pen. It took 2-3 training sessions to train the desired behavior in all pigs.

After the acclimatization period, measurements were started at 5 weeks of age. After 10 weeks the pigs were euthanized. The piglets were sedated using a 2 mg/kg intramuscular injection of Azaperone (Stresnil, Elanco Animal Health, Greenfield, USA). When the piglets were sufficiently sedated (no reaction to touch) they were euthanized by intracardial injection of 200 mg/kg Pentobarbital (Euthanimal, Alfasan, Woerden, The Netherlands). After euthanasia, the piglets were transported to the Department of Pathobiology of the Faculty of Veterinary Medicine of Utrecht University. Gross pathology was performed to confirm that the piglets were healthy at the time of death. Moreover, specific attention was paid to the limb joints. They were opened and inspected for any macroscopic signs of joint disease.

### Pressure mat analysis

Pigs were tested in the order they presented themselves, to minimize handling-associated stress. This appeared to be a highly stable order over the study weeks.

To perform the pressure mat analysis, the pigs were individually let out of their pen. They walked freely to the testing area. When they entered the holding pen, the area was closed and testing started. Two researchers, one in each of the holding pens, rewarded the pig only when it performed a correct run (crossing the entire length of the runway at the trot without stopping). If the run was not correct, the pig received no reward. If the pig still had not performed a correct run after 10 trials, it was placed back in the home pen and tested again one hour later. This occurred only once during the complete experiment.

A correct run had to fulfill the following additional criteria to be considered valid and to be included in the study: the pig had to trot the entire length of the runway at a visually steady pace in a straight line and looking straight ahead. These criteria were judged by two observers. At least 3 valid runs per pig were collected. Velocity was recorded by the pressure mat. All analyzed variables were automatically generated by the software.

### Data analysis

Claw strikes from the 3 valid runs were manually assigned to left fore (LF), right fore (RF), left hind (LH) and right hind (RH) limb. PVF (N), LR (N/s), VI (Ns) and PVP (N/cm^2^) were normalized to body mass (nPVF, nLR, nVI, nPVP). For every pig, mean nPVF, nLR, nVI and nPVP were calculated for each set of 3 valid runs. To allow comparison with data published by others [28,32,38-40], PVF and VI were also represented as percentage of bodyweight (% BW). For each run, the PVF was used to calculate the distribution of bodyweight over the four legs using the following formula:

$$\frac{\mathrm{PVF}\;\mathrm{of}\;\mathrm{the}\;\mathrm{limb}}{\mathrm{total}\;\mathrm{PVF}\;\mathrm{of}\;\mathrm{the}\;\mathrm{four}\;\mathrm{limbs}}*100$$

Fore and hind limb asymmetry indices (ASI) of all variables were calculated using the following formula [48]:

$$\frac{L-R}{0.5\left(L+R\right)}*100$$

According to this method, a value of 0% indicates perfect contra-lateral symmetry, whereas positive or negative values indicate relatively higher loading of the left or right limb, respectively. Possible values range from -200% to 200%.

For further statistical analysis, the absolute value of the ASI’s was used, removing the distinction between right- or left-sided asymmetry.

### Statistics

A linear mixed effects model was used to evaluate the effect of week, limb (left vs. right and fore vs. hind) as fixed factors and velocity as covariate on nPVF, nLR, nVI, nPVP and their ASI’s. nLR, nVI and nPVP were log-transformed, and square root transformation of ASI’s was used to meet normality assumptions. Data were analyzed using SPSS statistics 20 (IBM) and R 2.15 (R foundation for statistical computing) with Bonferroni-corrected statistical significance set at p *<* 0.05. In order to assess variability between runs of a pig on the same day, intra-class correlations (ICC) were calculated and interpreted according to Shrout and Fleiss [49]. The data are presented as means ± standard error of mean (SEM).

## Competing interests

The authors declare that they have no financial or non-financial competing interests.

## Authors’ contributions

EM contributed to study design and data collection, analyzed the data and drafted the manuscript. CB contributed to study design and data collection. MO provided advice on pressure mat data collection and analysis, and critically revised the manuscript. FJS aided in writing the first draft of the manuscript, provided advice on data analysis and critically revised the manuscript. WB contributed to study design, provided advice on automated gait analysis and critically revised the manuscript. AvN critically revised the manuscript. All authors read and approved the final manuscript.
